# Supplementary material for: Pulmonary arterial hypertension induces a metabolic and inflammatory hepatopathy
Source: J Clin Invest. 2026 May 28;136(13):e201862. doi: 10.1172/JCI201862 (PMC13318111; doi:10.1172/JCI201862)
Supplement: Supplemental data [file jci-136-201862-s134.pdf]

Supplemental Figure 1: Schematic representation of single nucleus RNA-sequencing workflow and data processing pipeline.

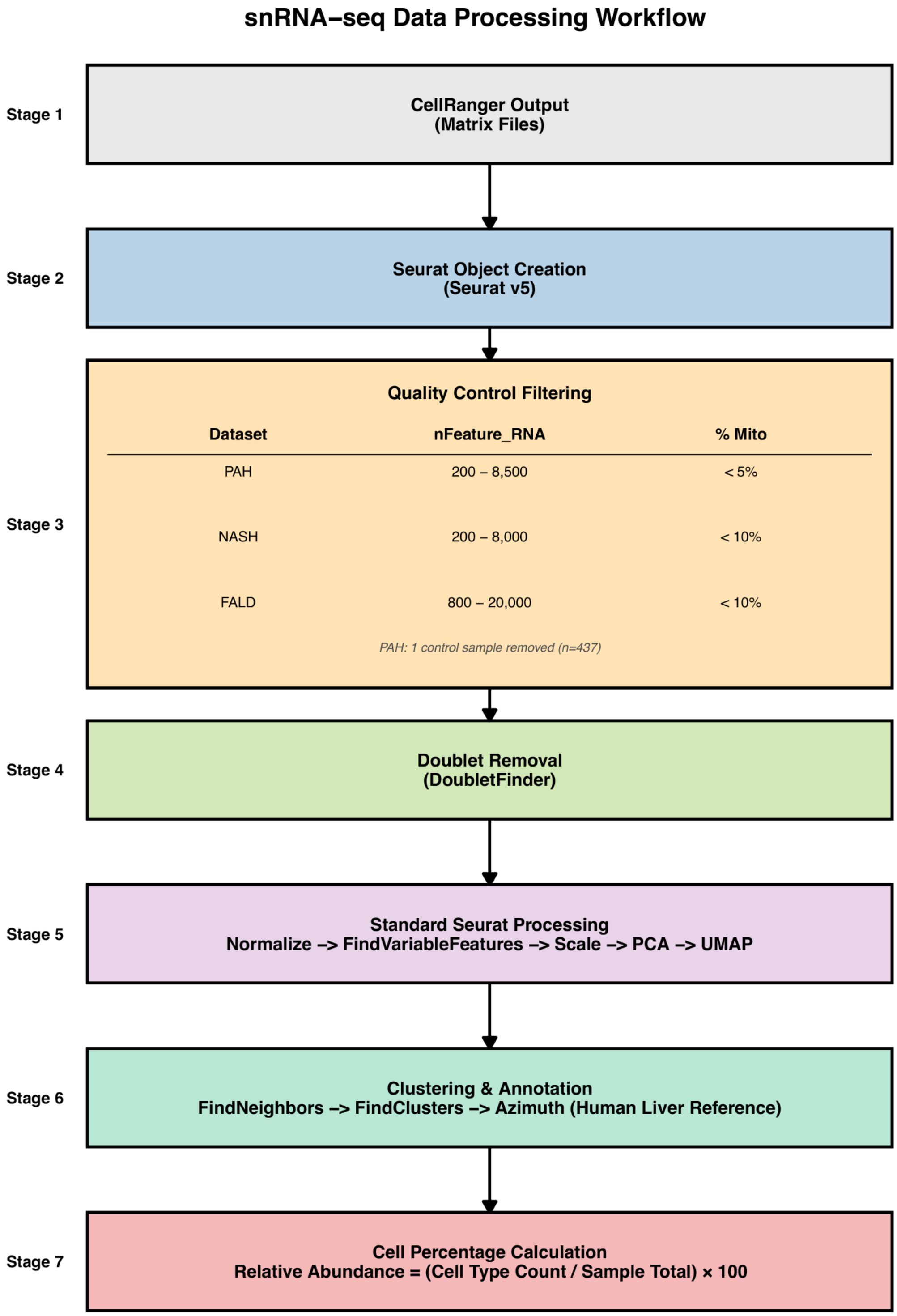

Supplemental Figure 2: Violin plots displaying key snRNA-seq quality control metrics across datasets, including total reads per nuclei, detected genes per cell, percent mitochondrial reads, and percent ribosomal reads. Median values are displayed and error bars represent medians with interquartile range.

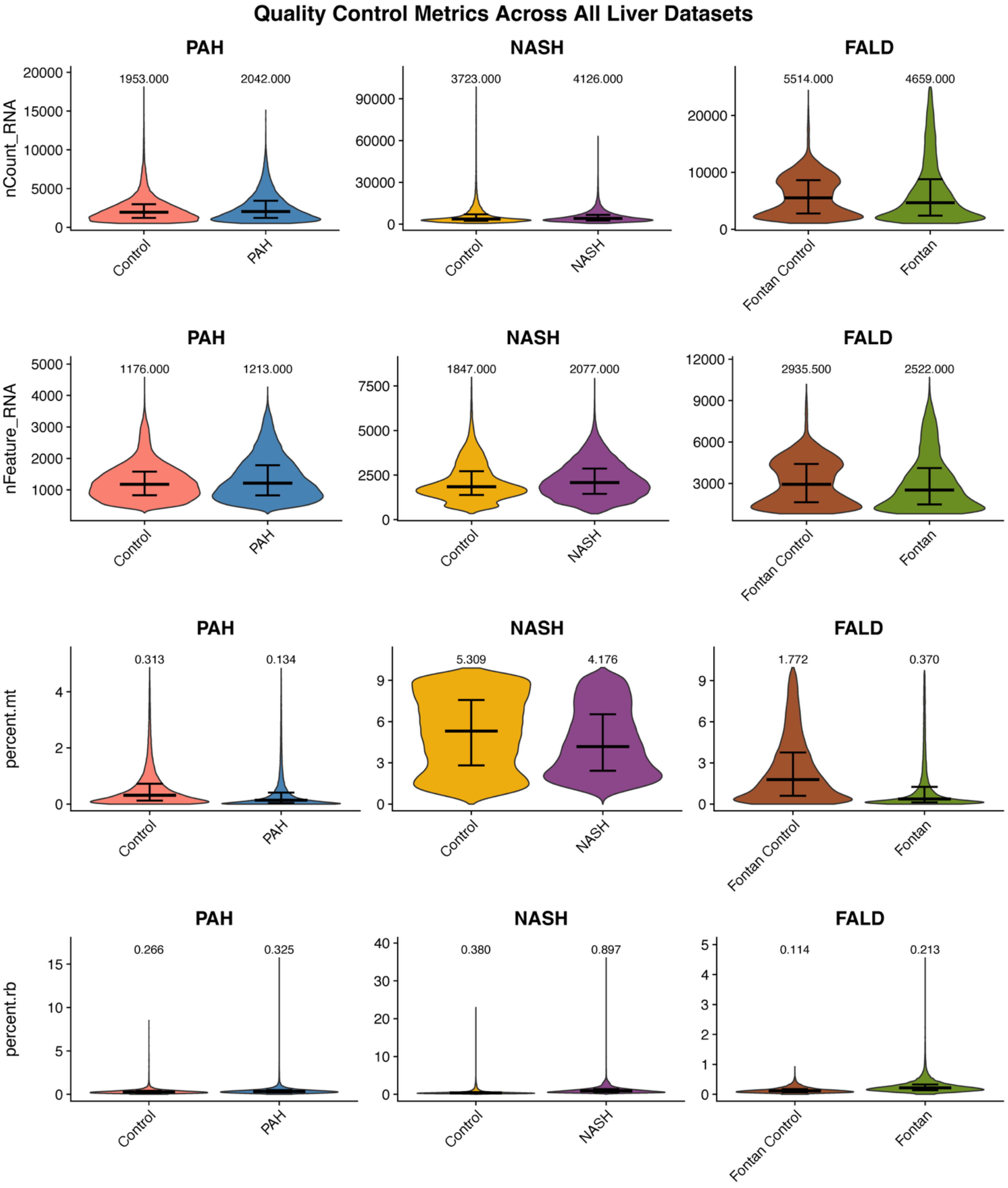

Supplemental Figure 3: Sample-specific relative abundance values across cell types in PAH, NASH, and FALD datasets. (A) Cell-type relative abundances in control and PAH samples. Error bars represent median values with range. (B) Cell-type relative abundances in control and NASH samples. Error bars represent median values with range. (C) Cell-type relative abundances in control and FALD samples. Error bars represent median values with range.

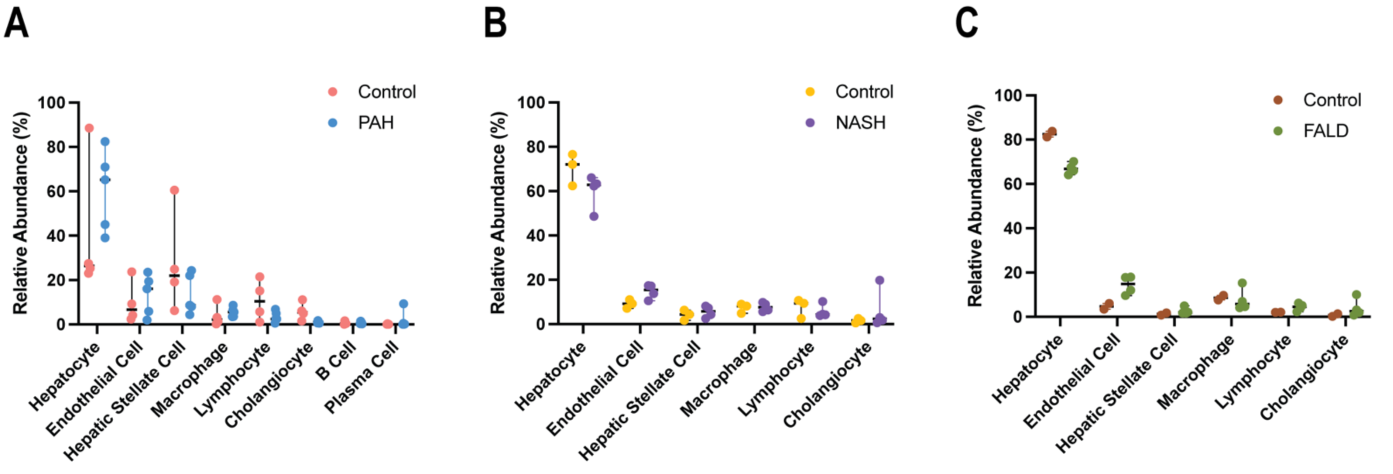

Supplemental Figure 4: KEGG and Wiki pathway analysis of enriched and suppressed DEGs from PAH, NASH, and FALD hepatocytes relative to their respective controls.

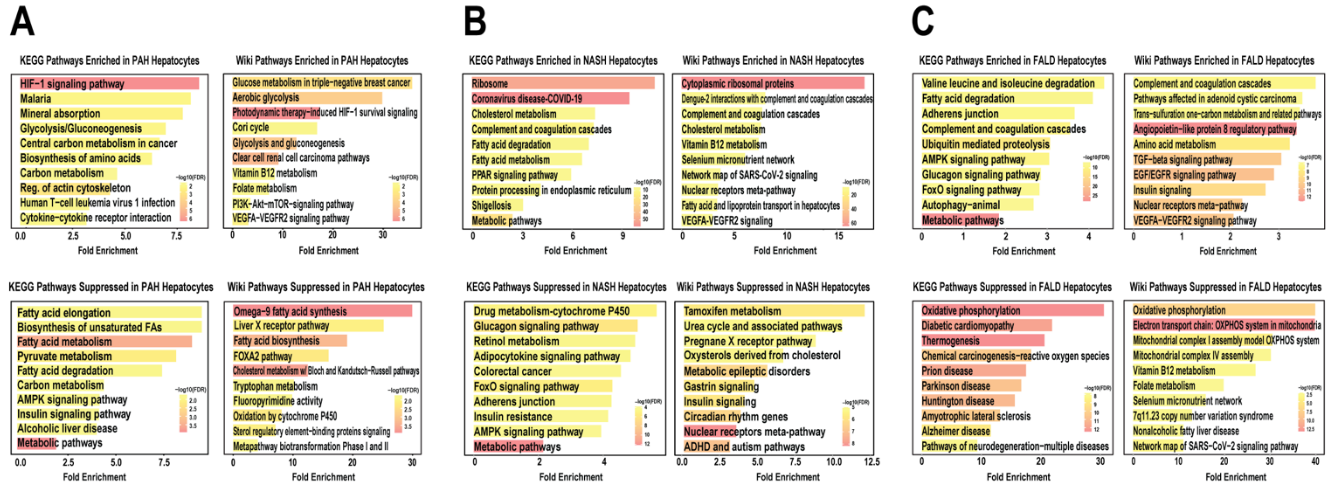

Supplemental Figure 5: KEGG and Wiki pathway analysis of enriched and suppressed DEGs from PAH, NASH, and FALD endothelial cells relative to their respective controls.

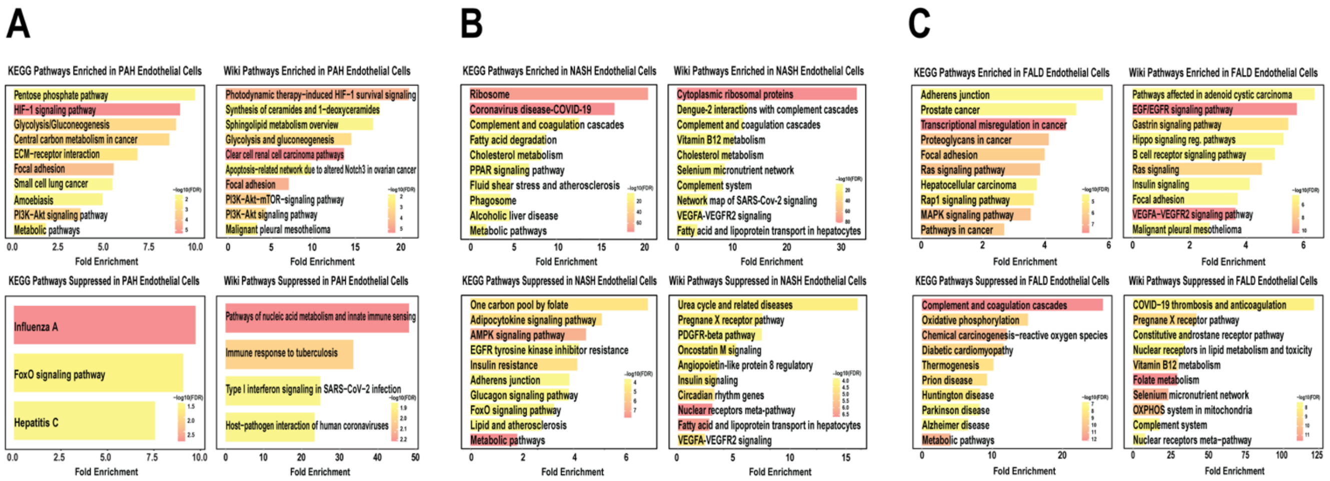

Supplemental Figure 6: KEGG and Wiki pathway analysis of enriched and suppressed DEGs from PAH, NASH, and FALD hepatic stellate cells relative to their respective controls.

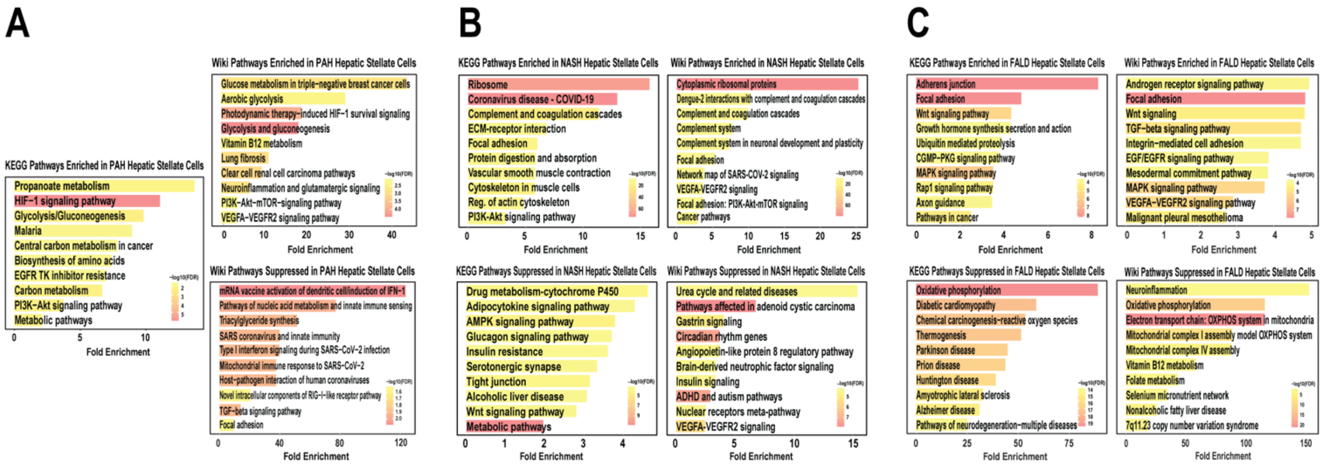

Supplemental Figure 7: Total hepatic fibrosis was elevated in PAH livers and associated with hepatic stellate cell HIF-1 signaling. (A) Representative whole-liver images and quantification of trichrome total fibrosis (%) staining in control (n=4) and PAH (n=5) livers. Error bars represent median values with range, and the *p*-value was calculated by Mann-Whitney test. (B) Linear regression analysis showing the association between total fibrosis (%) and hepatic stellate cell HIF-1 pseudobulked module scores in control (black) and PAH (red) patients. Dotted lines display 95% confidence intervals.

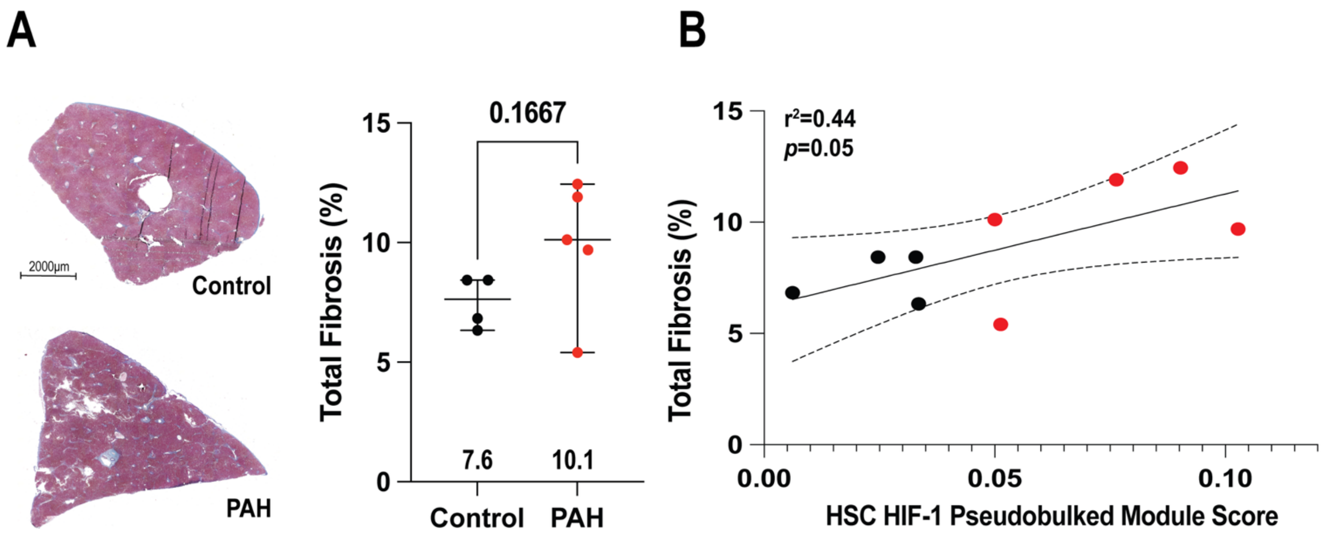

Supplemental Figure 8: KEGG and Wiki pathway analysis of enriched and suppressed DEGs from PAH, NASH, and FALD macrophages relative to their respective controls.

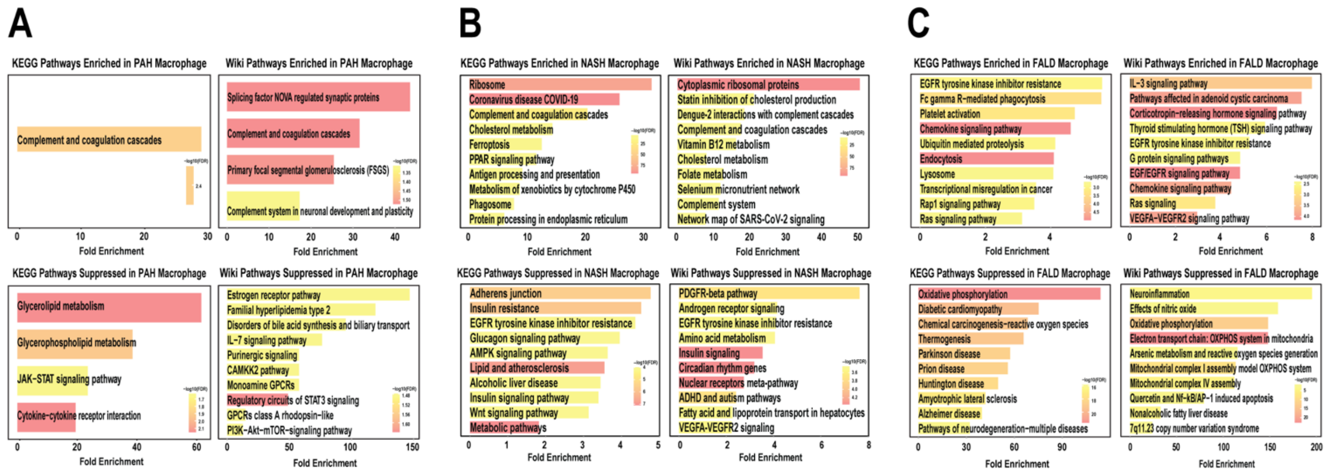

Supplemental Figure 9: Correlational heatmapping identified relationships between right ventricular function and HIF-1 signaling in hepatocytes, endothelial cells, and hepatic stellate cells. (A) Heatmap displaying Pearson correlation coefficients between clinical markers of right ventricular failure/PAH severity and cell type-specific HIF-1 pseudobulked module scores in hepatocytes, endothelial cells, and hepatic stellate cells in PAH patients (n=5). Pearson correlations were computed using pairwise complete observations; the effective n per variable pair ranged from 4 to 5 depending on clinical data availability. (B) Correlation coefficients (r) and corresponding *p*-values for the top five associations for each cell type-specific HIF-1 module score.

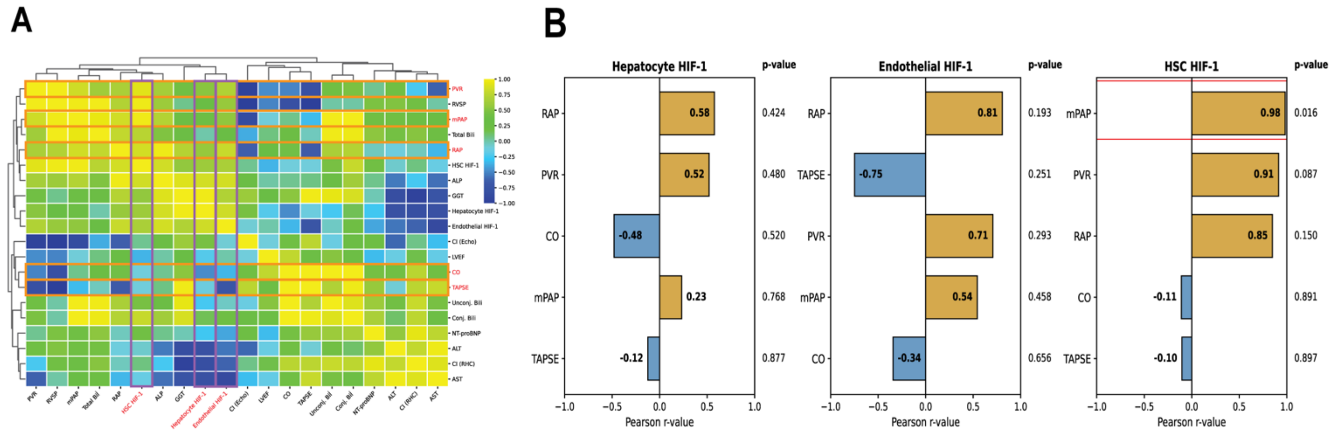

Supplemental Figure 10: Hepatic stellate cell mechanosensitive and inflammatory signaling associate with HIF-1 activation. (A) HSC PIEZO1 expression in control (n=4) and PAH (n=5) livers and its relationship with HSC HIF-1 pseudobulked module scores. Dot plots display median with range (Mann-Whitney test) and correlation plots show linear regression with 95% confidence intervals. **(B)** HSC IL-6 expression in control (n=4) and PAH (n=5) livers and its associations with PIEZO1 expression and HIF-1 signaling. Dot plots display median with range (Mann-Whitney test) and correlation plots show linear regression with 95% confidence intervals. **(C)** Schematic illustrating the proposed mechanism linking HSC activation to downstream HIF-1 signaling.

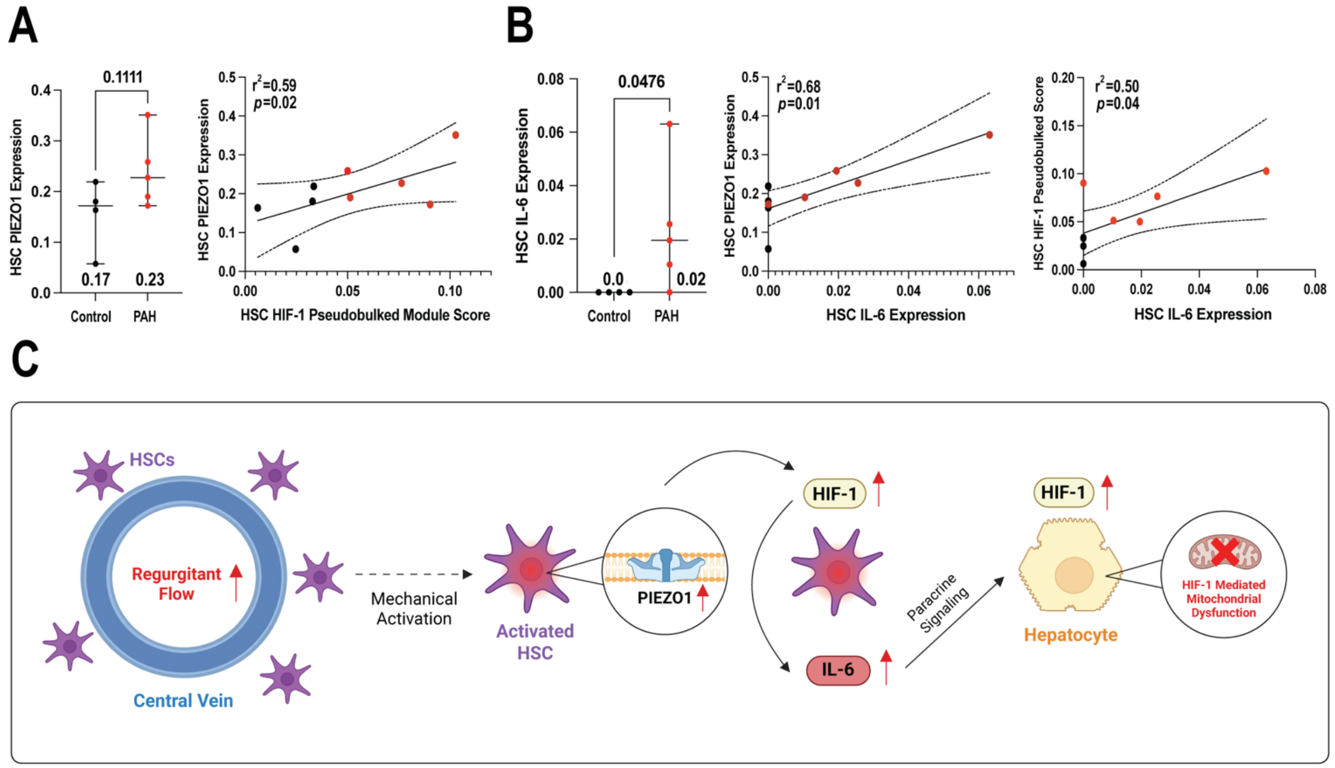

Supplemental Figure 11: Monocrotaline (MCT)-treated rodent livers exhibit trends toward reduced fatty acid metabolism and oxidative phosphorylation, consistent with some of the metabolic alterations observed in human PAH livers. Error bars represent median with range, and *p*-values were calculated using Mann-Whitney tests.

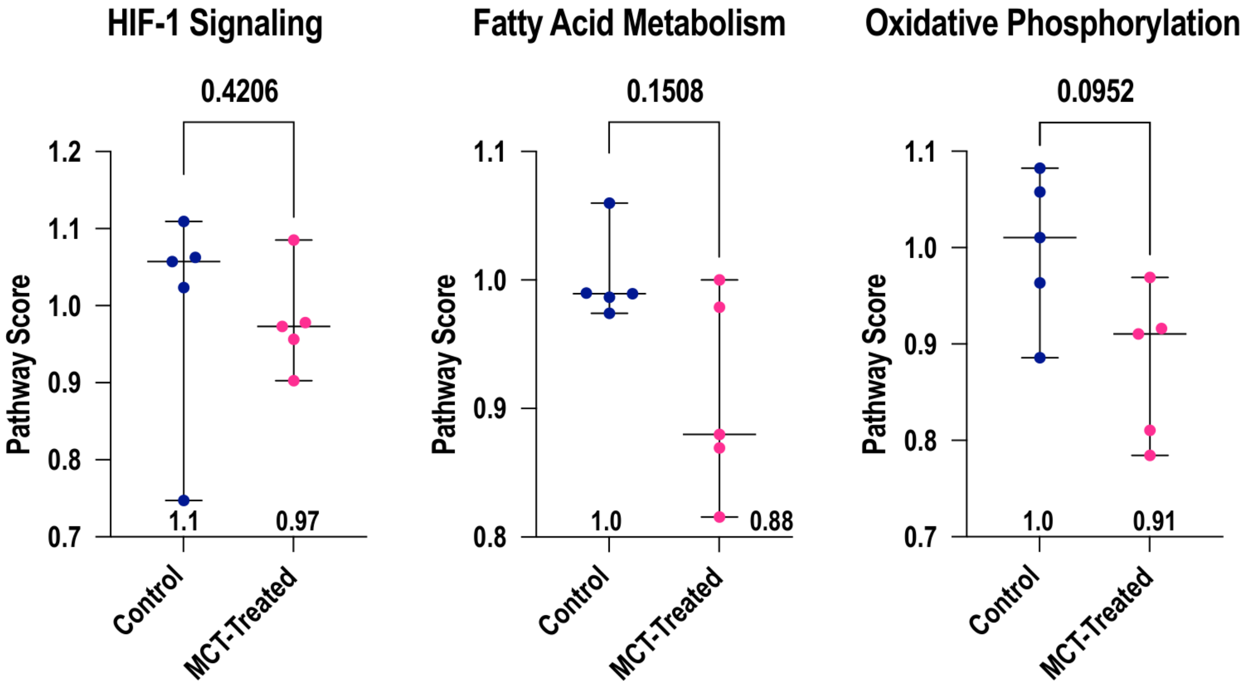

Supplemental video 1
Video displaying alpha smooth muscle actin staining (yellow) surrounding a hepatic central vein in a control patient on IHC.

Supplemental video 2
Video displaying alpha smooth muscle actin staining (yellow) surrounding a hepatic central vein in a PAH patient on IHC.
